# Development of a predictive model for risk stratification of acute kidney injury in patients undergoing cytoreductive surgery with hyperthermic intraperitoneal chemotherapy

**DOI:** 10.1038/s41598-024-54979-w

**Published:** 2024-03-19

**Authors:** Martin Krause, Soraya Mehdipour, Jula Veerapong, Joel M. Baumgartner, Andrew M. Lowy, Rodney A. Gabriel

**Affiliations:** 1 Division of Perioperative Informatics, Department of Anesthesiology, University of California San Diego, 200 West Arbor Drive, San Diego, CA 80203 USA; 2 Division of Surgical Oncology, Department of Surgery, University of California San Diego, San Diego, CA USA

**Keywords:** Intraperitoneal carcinomatosis, Acute kidney injury, Hyperthermic intraperitoneal chemotherapy, Predictive model, Acute kidney injury, Risk factors, Surgical oncology

## Abstract

Acute kidney injury (AKI) following hyperthermic intraperitoneal chemotherapy (HIPEC) is common. Identifying patients at risk could have implications for surgical and anesthetic management. We aimed to develop a predictive model that could predict AKI based on patients’ preoperative characteristics and intraperitoneal chemotherapy regimen. We retrospectively gathered data of adult patients undergoing HIPEC at our health system between November 2013 and April 2022. Next, we developed a model predicting postoperative AKI using multivariable logistic regression and calculated the performance of the model (area under the receiver operating characteristics curve [AUC]) via tenfold cross-validation. A total of 412 patients were included, of which 36 (8.7%) developed postoperative AKI. Based on our multivariable logistic regression model, multiple preoperative and intraoperative characteristics were associated with AKI. We included the total intraoperative cisplatin dose, body mass index, male sex, and preoperative hemoglobin level in the final model. The mean area under the receiver operating characteristics curve value was 0.82 (95% confidence interval 0.71–0.93). Our risk model predicted AKI with high accuracy in patients undergoing HIPEC in our institution. The external validity of our model should now be tested in independent and prospective patient cohorts.

## Introduction

Hyperthermic intraperitoneal chemotherapy (HIPEC) following cytoreductive therapy has become a common treatment for peritoneal metastases from mesothelioma, colorectal, gastric, appendiceal, ovarian, and primary peritoneal cancer^1^. Advantages include higher concentrations of heated chemotherapeutic agents with improved cytotoxicity and limited systemic side effects^2^.

Still, acute kidney injury (AKI) following HIPEC remains a known and common complication^3–7^, which is associated with increased length of stay and major postoperative morbidities^8^. While prior prediction models for AKI have focused on patients presenting for non-cardiac surgery^9,10^, patients undergoing HIPEC are exposed to more unique causes of AKI, including nephrotoxic chemotherapy agents^3–7^, excessive blood loss^5,8^, increased intraabdominal pressure during intraperitoneal perfusion^5^, and vasodilation related to induced hyperthermia intraoperatively^11^. Most recent literature focuses on intraoperative and postoperative interventions to reduce the risk of AKI following HIPEC^12,13^. But little is known about the ability to stratify the risk of postoperative AKI in this surgical patient population preoperatively.

Identifying patients at risk of AKI prior to HIPEC could have implications for the surgical and anesthetic plan. Modifiable risk factors can be optimized, nephrotoxic medication can be held prior to surgery, adjustments can be made to the type and dosing of intraperitoneal chemotherapeutic drugs, and alternative intraoperative hemodynamic monitoring techniques could be applied^14^. Thus, the objective of this study was to develop a predictive model for postoperative AKI using preoperative features such as demographics, chronic comorbidities, preoperative laboratory tests, home medications, and the chosen chemotherapy agent.

## Methods

Our study was approved by the University of California San Diego Institutional Review Board (IRB number 804874) and was carried out in accordance with the principles of the Declaration of Helsinki^15^. All methods were performed in accordance with the relevant guidelines and regulations. This study followed the Transparent Reporting of a multivariable prediction model for Individual Prognosis Or Diagnosis (TRIPOD) recommendations^16^. The aim of this study was to develop a predictive model for postoperative AKI based on data from the institution’s electronic health record (EHR) via manual chart review. Investigators were identified and authenticated via username and password, granting access to the institutional EHR. Password-protected and de-identified patient information was used for data analysis.

All adult patients that underwent HIPEC at the health system between November 2013 and April 2022 were eligible for inclusion. Exclusion criteria were age < 18 years old, patients who were scheduled but did not undergo intraoperative HIPEC, and patients on dialysis prior to surgery. The primary outcome of the study was postoperative AKI and defined as the difference between postoperative and preoperative creatinine closest to surgery ≥ 0.3 mg/dl within 48 postoperative hours or a quotient of postoperative and preoperative creatinine closest to surgery ≥ 1.5 within 7 postoperative days (KDIGO stage 1 or higher)^8,17^.

Covariates in the model were those known preoperatively and included: (1) patient demographics, (2) chronic comorbidities, (3) laboratory tests, (4) and medications prior to surgery. Patient demographics included: age in years, body mass index (BMI) in kg/m^2^ as continuous variables, and legal sex, race, and ethnicity as categorical variables. Chronic comorbidities were chosen from previously reported findings based on relevant pre-existing International Classification of Diseases codes (ICD-10) (Supplementary Table 1). These included the categorical variables: cardiac disease, asthma, chronic obstructive pulmonary disease (COPD), other pulmonary disease, hypertension, diabetes mellitus, renal disease, and liver disease. Laboratory tests were treated as categorical variables in the univariate and included preoperative creatinine, blood urea nitrogen (BUN), potassium, bicarbonate, total bilirubin, hemoglobin, platelet count, and glomerular filtration rate (GFR). Chronic kidney disease was defined as a preoperative estimated GFR < 60 ml/min/1.73 m^2^^18^. Anemia was defined as a hemoglobin level below 12 mg/dl for females and 13 mg/dl for males^9,19^. Categorical covariates on medications taken prior to surgery included renin inhibitors, nonsteroidal anti-inflammatory drugs (NSAIDs), nephrotoxic antiviral drugs, antibiotics, antifungals, chemotherapeutic agents, and antiepileptic drugs (Supplementary Table 2). AKI associated with intraperitoneal chemotherapy is dose-dependent^3^, and the intraoperative dosing of chemotherapeutic agents is standardized or otherwise determined preoperatively^20^. Therefore, we also considered the intraperitoneal dose of cisplatin, carboplatin, doxorubicin, gemcitabine, and mitomycin as continuous variables in our model.

R Statistical Programming Language (v4.4.2) was used for all statistical analyses. Initially, we compared statistical differences in each covariate in the two cohorts—patients who did not develop postoperative AKI *versus* patients that did develop postoperative AKI—using chi-squared test or Fisher’s exact test for categorical and Wilcoxon Rank Sum test or t-test for continuous variables, respectively. A P < 0.05 was considered statistically significant. To develop a predictive model, we utilized multivariable logistic regression with feature selection. The features selected for the final model were preoperatively known variables that had an association with the primary outcome on univariate logistic regression with P < 0.2 based on recommendations for prediction modeling^21^. After plotting the receiver operating characteristics curve (package pROC_1.18.5), model performance was measured by the area under the receiver operating characteristics curve (AUC). To calculate the AUC, we performed tenfold cross-validation, in which the entire dataset was split into 10 folds. Of those folds, nine served as the training set and one as the test set. The model was trained on the training set, and the AUC of that model was calculated on the test set. This was repeated until each fold served as the test set. The average AUC was then reported. Furthermore, to account for class imbalance, we applied Synthetic Minority Oversampling Technique (SMOTE) on the nine training folds (package smotefamily_1.3.1) when training the model for each iteration^22^. A dataset is considered imbalanced if there are large differences in the rate of negative versus positive outcomes. SMOTE takes samples of the feature space of the minority class (in this case, patients with postoperative AKI) and five of its nearest neighbors. Using this data, SMOTE then synthesizes new cases that combine features of the target case with features of its nearest neighbors by multiplying the difference between the examples by a number between 0 and 1. With the newly generated synthetic data for the minority class, the balance between the negative and positive classes becomes more balanced. Improving the balance between negative and positive classes may improve predictive performance of the models. Of note, SMOTE was only applied to training data and not test data.

We estimated the sample size needed for logistic regression using the equation N = 100 + EPV*i^23^, where the rule of event per variable (EPV) is 50 and i is the number of independent variables in the final model. The final model had four features; thus, the calculated sample size was 300.

### Ethics approval and consent to participate

The Institutional Review Board was approved (IRB number 804874), and the requirement for informed consent was waived.

## Results

During the study period, there were 556 patients who were scheduled to undergo HIPEC at our institution. After exclusion of ineligible participants, the final study population consisted of 412 patients, of which 36 (8.7%) developed postoperative AKI. Between the two cohorts (no postoperative AKI *versus* AKI), there were statistically significant differences between the following covariates: White, Black, preoperative total bilirubin and creatinine levels, cisplatin, doxorubicin, and mitomycin dose, crystalloids, packed red blood cell (PRBC) transfusions, and estimated blood loss (Table 1).

**Table 1:** Patient and demographic characteristics.

Characteristic	No acute kidney injury (N = 376)	Acute kidney injury (N = 36)	p-value
Preoperative characteristics			
Age (years)—Median (IQR)	55.00 (16.00)	59.50 (23.25)	0.242
BMI (kg/m^2^)—Median (IQR)	25.80 (6.52)	28.25 (8.07)	0.064
Male—no. (%)	159 (42)	20 (56)	0.174
Hispanic, Latino (a), Spanish origin—no. (%)	59 (16)	5 (14)	0.965
Race—no. (%)			
White	289 (77)	19 (53)	0.003
Black	3 (1)	6 (17)	< 0.001
Asian	24 (6)	3 (8)	0.720
Other	60 (16)	8 (22)	0.464
CHF—no. (%)	0 (0)	1 (3)	0.087
CAD—no. (%)	2 (1)	1 (3)	0.240
HTN—no. (%)	128 (34)	16 (44)	0.286
Other cardiac disease—no. (%)	17 (5)	1 (3)	1.000
Asthma—no. (%)	22 (6)	2 (6)	1.000
COPD—no. (%)	2 (1)	0 (0)	1.000
Other lung disease—no. (%)	2 (1)	1 (3)	0.240
Renal disease—no. (%)	9 (2)	1 (3)	0.604
Chronic kidney disease—no (%)	30 (8)	3 (8)	0.942
Liver disease—no. (%)	8 (2)	0 (0)	1.000
Diabetes mellitus—no. (%)	31 (8)	6 (17)	0.119
Anemia—no. (%)	106 (28)	21 (58)	< 0.001
Creatinine (mg/dl)—Median (IQR)	0.80 (0.24)	0.78 (0.37)	0.976
BUN (mg/dl)—Median (IQR)	14.00 (6.00)	14.50 (6.00)	0.610
Bicarbonate (mg/dl)—Median (IQR)	26.00 (3.00)	26.00 (3.00)	0.796
Total bilirubin (mg/dl)—Median (IQR)	0.34 (0.27)	0.28 (0.16)	0.047
Potassium (mg/dl)—Median (IQR)	4.30 (0.50)	4.20 (0.53)	0.603
Hemoglobin (mg/dl)—Median (IQR)	13.10 (2.20)	12.30 (2.50)	0.004
Renin inhibitor—no. (%)	78 (21)	8 (22)	1.000
NSAID—no. (%)	31 (8)	6 (17)	0.119
Nephrotoxic antiviral—no. (%)	5 (1)	1 (3)	0.424
Nephrotoxic antiepileptic– no. (%)	2 (1)	0 (0)	1.000
Nephrotoxic chemotherapeutic—no. (%)	3 (1)	0 (0)	1.000
Intraoperative characteristics			
Estimated blood loss (ml)—Median (IQR)	150.00 (200.00)	250.00 (612.50)	0.042
Crystalloids (ml)—Median (IQR)	4300.00 (2500.00)	5000.00 (2575.00)	0.021
Colloids (ml)—Median (IQR)	1500.00 (1000.00)	1500.00 (1500.00)	0.121
PRBC (units)—Median (IQR)	0 (0)	0 (1.00)	0.002
FFP (units)—Median (IQR)	0 (0)	0 (0)	0.092
Platelet transfusions (units)—Median (IQR)	0 (0)	0 (0)	0.364
Mitomycin (mg)—Mean (SD)	35.16 (11.82)	18.61 (21.00)	< 0.001
Cisplatin (mg)—Mean (SD)	7.30 (26.60)	54.56 (54.12)	< 0.001
Carboplatin (mg)—Mean (SD)	10.85 (148.99)	0 (0)	0.159
Doxorubicin (mg)—Mean (SD)	1.64 (6.58)	12.47 (14.76)	< 0.001
Gemcitabine (mg)—Mean (SD)	6.62 (92.43)	0 (0)	0.166
Maximum temperature (°C)—Median (IQR)	41.50 (1.00)	41.50 (1.00)	0.380
Perfusion time (minutes)—Median (IQR)	90.00 (0)	90.00 (0)	0.666
Phenylephrine (mcg)—Mean (SD)	4810.74 (6679.12)	6625.89 (7883.35)	0.189
Ephedrine (mg)—Mean (SD)	14.00 (16.78)	18.89 (19.72)	0.158
Vasopressin (units)—Mean (SD)	0.37 (1.72)	0.49(1.76)	0.706
Epinephrine (mcg)—Mean (SD)	3.25 (52.96)	0 (0)	0.235
Norepinephrine (mcg)—Mean (SD)	3.81 (53.03)	0 (0)	0.165
Dobutamine (mcg)—Mean (SD)	0.02 (0.45)	0 (0)	0.318
Dopamine (mcg)—Mean (SD)	0.08 (1.50)	0 (0)	0.318
Postoperative characteristics			
30-day mortality—no. (%)	1 (0)	0 (0)	1.000

Comparison of variables in patients without AKI *versus* patients with AKI. Variables were compared between patients without and with acute kidney injury with Fisher’s Exact test and Chi-Square test for categorical variables and Wilcoxon Rank Sum test and t-test for continuous variables. We reported the number and percentage of patients for categorical variables and median values and the interquartile range (IQR) for continuous variables. For dosing, we reported mean values and standard deviation (SD). Variables searched for but not present were not reported.

*BMI* body mass index, *CAD* coronary artery disease, *CHF* congestive heart failure, *HTN* hypertension, *COPD* chronic obstructive pulmonary disease. *NSAID* non-steroidal anti-inflammatory drug, *PRBC* packed red blood cells, *FFP* fresh frozen plasma.

Male sex, BMI, White, Black, preoperative hemoglobin and total bilirubin levels, coronary artery disease, diabetes mellitus, lung disease other than COPD or asthma, NSAIDs taken prior to surgery, estimated blood loss, crystalloids, colloids, PRBC and platelet transfusions, the total intraoperative dose of phenylephrine and ephedrine as well as the total intraoperative dose of mitomycin, cisplatin, and doxorubicin had an association with the primary outcome in the univariate regression modeling AKI (Table 2).

**Table 2:** Univariate regression modeling.

Characteristic	Odds ratio	95% confidence limits	p-value
Preoperative characteristics			
Age	1.007	0.979–1.037	0.617
BMI	1.065	1.009–1.125	0.023
Male	1.706	0.857–3.396	0.128
Hispanic, Latino(a), Spanish origin—no. (%)	0.867	0.324–2.320	0.776
Race			
White	0.336	0.168–0.675	0.002
Black	24.867	5.921–104.426	< 0.001
Asian	1.333	0.381–4.664	0.652
Other	1.505	0.654–3.461	0.336
CHF	Infinity	0.000-infinity	0.985
CAD	5.343	0.473–60.406	0.176
HTN	1.550	0.777–3.094	0.214
Other cardiac disease	0.603	0.078–4.670	0.628
Asthma	0.947	0.2134–4.199	0.942
COPD	0.000	0.000-infinity	0.990
Other lung disease	5.343	0.473–60.406	0.176
Renal disease	1.165	0.143–9.466	0.886
Chronic kidney disease	1.048	0.304–3.621	0.940
Liver disease	0.000	0.000-infinity	0.987
Diabetes mellitus	2.226	0.860–5.758	0.099
Anemia	3.566	1.772–7.178	< 0.001
Creatinine	1.428	0.323–6.308	0.638
BUN	1.022	0.953–1.095	0.546
Bicarbonate	1.007	0.889–1.141	0.912
Total bilirubin	0.183	0.0256–1.306	0.090
Potassium	0.760	0.322–1.794	0.531
Hemoglobin	0.765	0.627–0.934	0.008
Renin inhibitor	1.092	0.479–2.489	0.835
NSAID	2.226	0.860–5.758	0.099
Nephrotoxic antiviral	2.120	0.241–18.657	0.498
Nephrotoxic antiepileptic	0.000	0.000-infinity	0.990
Nephrotoxic chemotherapeutic	0.000	0.000-infinity	0.987
Intraoperative characteristics			
Estimated blood loss	1.001	1.000–1.001	0.030
Crystalloids	1.000	1.000–1.000	0.029
Colloids	1.000	1.000–1.000	0.042
PRBC	1.173	1.020–1.349	0.026
FFP	1.186	0.956–1.472	0.121
Platelet transfusions	1.656	0.911–3.012	0.098
Mitomycin dose	0.937	0.918–0.957	< 0.001
Cisplatin dose	1.025	1.017–1.033	< 0.001
Carboplatin dose	0.993	0.365–2.703	0.990
Doxorubicin dose	1.092	1.062–1.123	< 0.001
Gemcitabine dose	0.988	0.181–5.401	0.989
Maximum temperature	1.444	0.650–3.210	0.367
Perfusion time	0.047	0.000-infinity	0.989
Phenylephrine dose	1.000	1.000–1.000	0.131
Ephedrine dose	1.014	0.997–1.031	0.105
Vasopressin dose	1.035	0.869–1.232	0.699
Epinephrine dose	0.286	0.000-infinity	0.990
Norepinephrine dose	0.979	0.052–18.602	0.989
Dobutamine dose	0.245	0.000-infinity	0.989
Dopamine dose	0.653	0.000-infinity	0.989
Postoperative characteristics			
30-day mortality	0.000	0.000-infinity	0.989

Odds ratio, 95% confidence intervals, and p-values of univariate regression modeling AKI for each variable.

*BMI* body mass index, *CAD* coronary artery disease, *CHF* congestive heart failure, *HTN* hypertension, *COPD* chronic obstructive pulmonary disease. *NSAID* non-steroidal anti-inflammatory drug, *PRBC* packed red blood cells, *FFP* fresh frozen plasma.

**Figure 1:**
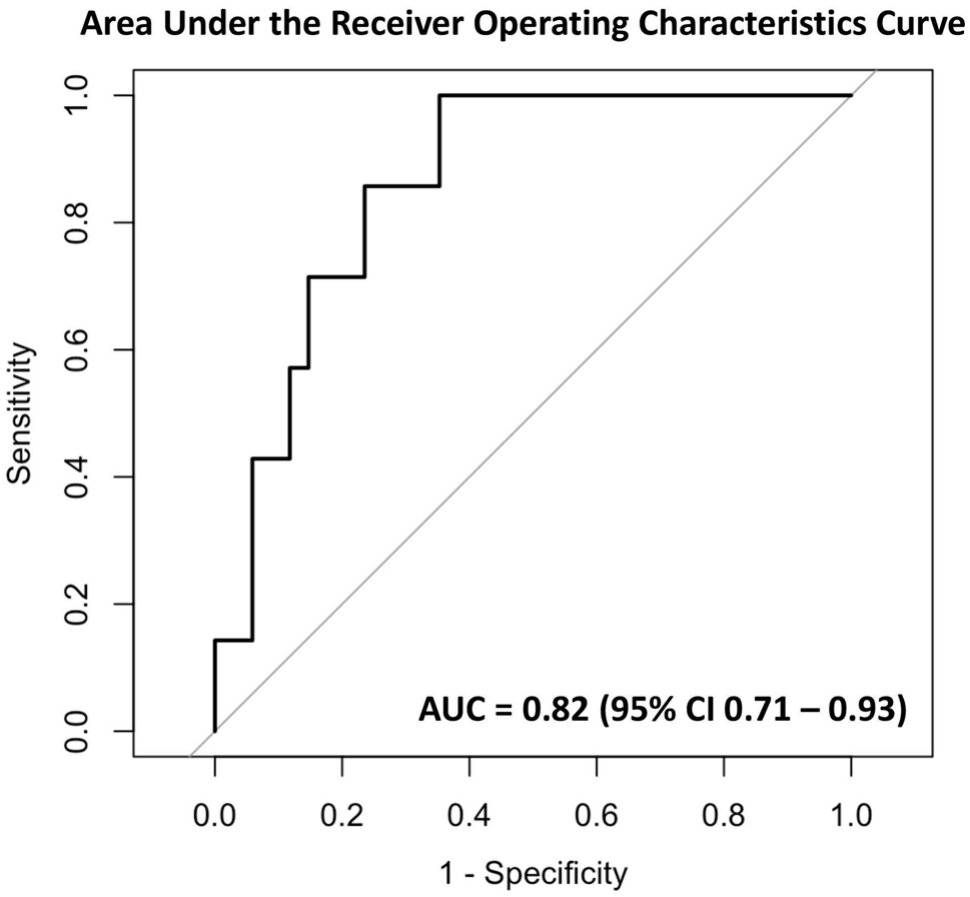
Plot illustrating mean (95% confidence intervals) area under the receiver operating characteristics curve from cross-validation.

For our final model, multivariable logistic regression was utilized with the features BMI (OR 1.073, 95% CI 1.006–1.144, P = 0.031), preoperative hemoglobin level (OR 0.738, 95% CI 0.589–0.925, P = 0.008), male sex (2.033, 95% CI 0.905–4.567, P = 0.086), and intraperitoneal cisplatin (OR 1.023, 95% CI 1.015–1.031, P < 0.001) (Table 3). In Fig. 1, a plot illustrates the mean AUC (0.82, 95% confidence interval 0.71–0.93) from cross-validation.

**Table 3:** Multivariable regression modeling.

Characteristic	Odds ratio	95% confidence interval	p-value
BMI	1.073	1.006–1.144	0.031
male	2.033	0.905–4.567	0.086
preoperative hemoglobin	0.738	0.589–0.925	0.008
cisplatin dose	1.023	1.015–1.031	< 0.001

Odds ratios, 95% confidence intervals, and p-values of the multivariable regression modeling AKI for each variable that was included in the final model.

*BMI* body mass index.

Based on the logistic regression model for predicting postoperative AKI in patients undergoing HIPEC surgery, the probability of the outcome may be calculated with the following equation:

$$P\,\left( {{\text{postoperative}}\,{\text{ AKI}}} \right)\, \, = { 1}/\left( {{1} + e^{{ - ( - {1}.{29} + 0.0{7}*{\text{BMI}} - 0.{3}*{\text{preoperative hemoglobin}} + 0.0{2}*{\text{cisplatin dose}} + 0.{71}*{\text{male sex}})}} } \right).$$

Thus, the probability of postoperative AKI can be estimated and compared to other patients with different combinations of risk factors. For example, if a male patient had a BMI of 30 kg/m^2^, preoperative hemoglobin of 16 g/dl, and did not receive cisplatin, the probability of AKI would be:

$$P\left( {\text{postoperative AKI}} \right) \, = { 1}/\left( {{1} + e^{{ - ( - {1}.{29} + 0.0{7}*{3}0 - 0.{3}*{16} + 0.0{2}*0 + 0.{71})}} } \right) \, = \, 0.0{36 }\left( {{\text{or 3}}.{6}\% } \right).$$

The same patient, but with a preoperative hemoglobin of 8, would have the following probability:

$$P\left( {\text{postoperative AKI}} \right) \, = { 1}/\left( {{1} + e^{{ - ( - {1}.{29} + 0.0{7}*{3}0 - 0.{3}*{8} + 0.0{2}*0 + 0.{71})}} } \right) \, = \, 0.{29 }\left( {{\text{or 29}}\% } \right).$$

## Discussion

In our study, 8.7% of patients undergoing HIPEC developed postoperative AKI. Our primary outcome was associated with multiple preoperatively known variables and intraoperatively administered chemotherapeutic agents. The final predictive model for AKI included BMI, hemoglobin, male sex, and total dose of intraperitoneal cisplatin in our final model, which accurately predicted postoperative AKI (AUC of 0.82).

Obesity is a known independent risk factor of perioperative renal dysfunction, likely explained by proinflammatory, hemodynamic, and pharmacokinetic factors unique to obese patients^24,25^. Not surprisingly, low hemoglobin levels preoperatively are independent predictors of postoperative AKI in cardiac and non-cardiac surgery^9,26^. Male sex is associated with postoperative AKI and forms part of multiple validated risk scores^9,27^. Cisplatin is a known nephrotoxic drug and strongly associated with perioperative AKI following intraperitoneal administration^3–7^.

Prior univariate and multivariate regression analyses of patients undergoing HIPEC have found associations between postoperative AKI and a multitude of variables such as age, obesity, preoperative creatinine and urea levels, intraperitoneal cisplatin, excessive blood loss, low perioperative diuresis, and the extent of peritoneal cancer^3–6,8^. Our retrospective study analyzed one of the most extensive data sets focusing on this surgical patient population. Prior retrospective analyses have focused on identifying different risk factors for postoperative AKI. This study developed a predictive model for postoperative renal injury following HIPEC and could, thus, be utilized to identify patients at risk and optimize these patients preoperatively.

In our study, 8.7% developed postoperative AKI, which is less common than previously reported. The incidence in other studies varies between 11.1 and 47.5% and is likely related to different guidelines on defining postoperative AKI and varying use of cisplatin^3–7,26^. As expected, our study found a dose-dependent association between cisplatin and AKI. Furthermore, the intraperitoneal dose of doxorubicin was also associated with AKI, previously only described for systemic administration^28,29^. However, doxorubicin was only administered in conjunction with cisplatin. We, therefore, did not include doxorubicin in our final model to avoid confounding.

Postoperative AKI is associated with increased length of stay and health care costs, chronic kidney disease, dialysis-dependence, and death^27^. Risk indices for perioperative AKI in non-cardiac surgery have been developed in the past, identifying similar predictors such as male sex and anemia, amongst others^9,30^. However, HIPEC exposes patients to unique risk factors, such as cisplatin^3–7^, and increased intraabdominal pressure possibly exacerbated by obesity^5^. Our model could predict postoperative AKI with high discriminatory ability based on only four predictors.

Identifying patients at risk of AKI prior to HIPEC could have implications to the surgical and anesthetic plan. The volume status of patients with risk factors such as male sex and high BMI can be optimized prior to surgery. When possible, nephrotoxic medication should be replaced by equally effective alternative drugs^14,17^. In patients at high risk for AKI, adjustments can be made to the type or dosing of intraperitoneal chemotherapeutic agent. Preexisting anemia can be corrected before proceeding with surgery^26^, preoperative weight loss in obese patients can be encouraged^31^, and nephroprotective measures such as invasive intraoperative hemodynamic monitoring could be applied to patients at risk^32^. In the immediate postoperative period, renin inhibitors and contrast should be avoided, and glucose levels should be well controlled^17^.

Our study has several limitations. First, retrospective data analyses, in general, are confounded by missed data or unaccounted confounding variables. We excluded patients with the most critical data missing, such as type and dosing of intraperitoneal chemotherapy, and imputed missing laboratory values by utilizing the cohort’s median. While data imputation can affect a model’s external validity, it was only implemented for missing preoperative bicarbonate and bilirubin levels, neither included in the final model. Second, we were constrained to the covariates, which were selected and collected from our institutional EHR. Diagnoses of comorbidities were binary and did not include stages of severity. Still, our data set was extensive including patient’s baseline laboratory data, which are often utilized to stage end-organ injury such as creatinine and total bilirubin for renal and liver dysfunction^17,33^. We intentionally limited the covariates in our model to preoperatively known data points allowing clinicians to adjust anesthetic and surgical plans. However, we decided to include the type and dosing of intraperitoneal chemotherapeutic agent as it is often chosen prior to surgery and a known contributor to nephrotoxicity^6^. Lastly, there was no separate external validation set for the study and hence, the reported accuracy of the model has limited generalizability. We, therefore, further need to conduct external validation using data outside of our institutional dataset. The discriminatory ability of our model might be lower in other patient populations from different geographic locations and socioeconomic backgrounds.

To conclude, our model was able to predict AKI within the first seven days postoperatively in patients undergoing HIPEC in our institution and provides the surgical and anesthesia team with a potentially helpful preoperative tool. Future trials must confirm the external validity of our model.

### Supplementary Information

Supplementary Table 1.

Supplementary Table 2.

## Data Availability

The datasets used and/or analyzed during the current study are available from the corresponding author on reasonable request.
