# Supplementary material for: Development of a predictive model for risk stratification of acute kidney injury in patients undergoing cytoreductive surgery with hyperthermic intraperitoneal chemotherapy
Source: Sci Rep. 2024 Mar 19;14:6630. doi: 10.1038/s41598-024-54979-w (PMC10951241; doi:10.1038/s41598-024-54979-w)
Supplement: Supplementary file 1 — Supplementary Table 1. [file 41598_2024_54979_MOESM1_ESM.docx]

| **Supplementary Table 1: ICD-10 codes** | | |
| --- | --- | --- |
| **ICD-10 code** | **Category** | **Diagnosis** |
| I01 | chronic cardiac disease | Rheumatic fever with heart involvement |
| I05 | chronic cardiac disease | Rheumatic mitral valve diseases |
| I06 | chronic cardiac disease | Rheumatic aortic valve diseases |
| I07 | chronic cardiac disease | Rheumatic tricuspid valve diseases |
| I08 | chronic cardiac disease | Multiple valve diseases |
| I09 | chronic cardiac disease | Other rheumatic heart diseases |
| I11 | chronic cardiac disease | Hypertensive heart disease |
| I13 | chronic cardiac disease | Hypertensive heart and chronic kidney disease |
| I25 | chronic cardiac disease | Chronic ischemic heart disease |
| I27 | chronic cardiac disease | Other pulmonary heart diseases |
| I27.82 | chronic cardiac disease | chronic pulmonary embolism |
| I31 | chronic cardiac disease | Other diseases of pericardium |
| I32 | chronic cardiac disease | Pericarditis in diseases classified elsewhere |
| I34 | chronic cardiac disease | Nonrheumatic mitral valve disorders |
| I35 | chronic cardiac disease | Nonrheumatic aortic valve disorders |
| I36 | chronic cardiac disease | Nonrheumatic tricuspid valve disorders |
| I37 | chronic cardiac disease | Nonrheumatic pulmonary valve disorders |
| I42 | chronic cardiac disease | Cardiomyopathy |
| I43 | chronic cardiac disease | Cardiomyopathy in diseases classified elsewhere |
| I44 | chronic cardiac disease | Atrioventricular and left bundle-branch block |
| I45 | chronic cardiac disease | Other conduction disorders |
| I46 | chronic cardiac disease | Cardiac arrest |
| I47 | chronic cardiac disease | Paroxysmal tachycardia |
| I48 | chronic cardiac disease | Atrial fibrillation and flutter |
| I49 | chronic cardiac disease | Other cardiac arrhythmias |
| I50 | chronic cardiac disease | Heart failure |
| I51 | chronic cardiac disease | Complications and ill-defined descriptions of heart disease |
| I52 | chronic cardiac disease | Other heart disorders in diseases classified elsewhere |
| J81 | chronic cardiac disease | Pulmonary edema |
| J40 | chronic pulmonary disease | Bronchitis, not specified as acute or chronic |
| J41 | chronic pulmonary disease | Simple and mucopurulent chronic bronchitis |
| J42 | chronic pulmonary disease | Unspecified chronic bronchitis |
| J43 | chronic pulmonary disease | Emphysema |
| J44 | chronic pulmonary disease | Other chronic obstructive pulmonary disease |
| J45 | chronic pulmonary disease | Asthma |
| J47 | chronic pulmonary disease | Bronchiectasis |
| J60 | chronic pulmonary disease | Coalworker's pneumoconiosis |
| J61 | chronic pulmonary disease | Pneumoconiosis due to asbestos and other mineral fibers |
| J62 | chronic pulmonary disease | Pneumoconiosis due to dust containing silica |
| J63 | chronic pulmonary disease | Pneumoconiosis due to other inorganic dusts |
| J64 | chronic pulmonary disease | Unspecified pneumoconiosis |
| J65 | chronic pulmonary disease | Pneumoconiosis associated with tuberculosis |
| J66 | chronic pulmonary disease | Airway disease due to specific organic dust |
| J67 | chronic pulmonary disease | Hypersensitivity pneumonitis due to organic dust |
| J68 | chronic pulmonary disease | Respiratory conditions due to inhalation of chemicals, gases, fumes and vapors |
| J69 | chronic pulmonary disease | Pneumonitis due to solids and liquids |
| J70 | chronic pulmonary disease | Respiratory conditions due to other external agents |
| J82 | chronic pulmonary disease | Pulmonary eosinophilia, not elsewhere classified |
| J84 | chronic pulmonary disease | Other interstitial pulmonary diseases |
| J90 | chronic pulmonary disease | Pleural effusion, not elsewhere classified |
| J91 | chronic pulmonary disease | Pleural effusion in conditions classified elsewhere |
| J92 | chronic pulmonary disease | Pleural plaque |
| J94 | chronic pulmonary disease | Other pleural conditions |
| J96 | chronic pulmonary disease | Respiratory failure, not elsewhere classified |
| J98 | chronic pulmonary disease | Other respiratory disorders |
| J99 | chronic pulmonary disease | Respiratory disorders in diseases classified elsewhere |
| N03 | chronic kidney disease | Chronic nephritic syndrome |
| N04 | chronic kidney disease | Nephrotic syndrome |
| N05 | chronic kidney disease | Unspecified nephritic syndrome |
| N06 | chronic kidney disease | Isolated proteinuria with specified morphological lesion |
| N07 | chronic kidney disease | Hereditary nephropathy, not elsewhere classified |
| N08 | chronic kidney disease | Glomerular disorders in diseases classified elsewhere |
| N11 | chronic kidney disease | Chronic tubulo-interstitial nephritis |
| N12 | chronic kidney disease | Tubulo-interstitial nephritis, not specified as acute or chronic |
| N13 | chronic kidney disease | Obstructive and reflux uropathy |
| N14 | chronic kidney disease | Drug- and heavy-metal-induced tubulo-interstitial and tubular conditions |
| N15 | chronic kidney disease | Other renal tubulo-interstitial diseases |
| N16 | chronic kidney disease | Renal tubulo-interstitial disorders in diseases classified elsewhere |
| N18 | chronic kidney disease | Chronic kidney disease (CKD) |
| N19 | chronic kidney disease | Unspecified kidney failure |
| N25 | chronic kidney disease | Disorders resulting from impaired renal tubular function |
| N26 | chronic kidney disease | Unspecified contracted kidney |
| N27 | chronic kidney disease | Small kidney of unknown cause |
| N28 | chronic kidney disease | Other disorders of kidney and ureter, not elsewhere classified |
| N29 | chronic kidney disease | Other disorders of kidney and ureter in diseases classified elsewhere |
| I13 | chronic kidney disease | Hypertensive heart and chronic kidney disease |
| K70 | chronic liver disease | Alcoholic liver disease |
| K71 | chronic liver disease | Toxic liver disease |
| K72 | chronic liver disease | Hepatic failure, not elsewhere classified |
| K73 | chronic liver disease | Chronic hepatitis, not elsewhere classified |
| K74 | chronic liver disease | Fibrosis and cirrhosis of liver |
| K75 | chronic liver disease | Other inflammatory liver diseases |
| K76 | chronic liver disease | Other diseases of liver |
| K77 | chronic liver disease | Liver disorders in diseases classified elsewhere |
| E08 | Diabetes mellitus | Diabetes mellitus due to underlying condition |
| E09 | Diabetes mellitus | Drug or chemical induced diabetes mellitus |
| E10 | Diabetes mellitus | Type 1 diabetes mellitus |
| E11 | Diabetes mellitus | Type 2 diabetes mellitus |
| E13 | Diabetes mellitus | Other specified diabetes mellitus |
| I10 | Hypertensive Diseases | essential hypertension |
| I15 | Hypertensive Diseases | secondary hypertension |
