# Supplementary material for: Development of a predictive model for risk stratification of acute kidney injury in patients undergoing cytoreductive surgery with hyperthermic intraperitoneal chemotherapy
Source: Sci Rep. 2024 Mar 19;14:6630. doi: 10.1038/s41598-024-54979-w (PMC10951241; doi:10.1038/s41598-024-54979-w)
Supplement: Supplementary file 2 — Supplementary Table 2. [file 41598_2024_54979_MOESM2_ESM.docx]

| Supplementary Table 2: nephrotoxic drugs | |
| --- | --- |
| characteristic | **drugs** |
| renin inhibitors | captopril, imidapril, zofenopril, candesartan, delapril, telmisartan, aliskiren, moexipril, enalapril, valsartan, fosinopril, irbesartan, perindopril, quinapril, ramipril, eprosartan, olmesartan, trandolapril, losartan, azilsartan, lisinopril, spirapril, benazepril, cilazapril, eplerenone, spirinolactone |
| nephrotoxic antivirals | acyclovir, cidofovir, gancyclovir, valacyclovir, valganciclovir |
| nephrotoxic antifungals | amphotericin B |
| nonsteroidal anti-inflammatory drugs | ibuprofen, naproxen, ketorolac, celecoxib, meloxicam, indomethacin, diclofenac |
| nephrotoxic chemotherapy or immunosuppressants | carboplatin, cisplatin, ifosfamide, methotrexate, sirolimus, tacrolimus, mesalamine, cyclosporine, sulfasalazine |
| nephrotoxic antipsychotics | lithium |
| nephrotoxic antibiotics | nafcillin, piperacillin/tazobactam, piperacillin, ticarcillin/clavulanic acid, tobramycin, vancomycin, gentamycin, dapsone, cefotaxime, ceftazidime, cefuroxime, colistimethate |
| nephrotoxic antiepileptics | topiramate, zonisamide |
